# The Role of Blood Oxygen Level Dependent Signal Variability in Pediatric Neuroscience: A Systematic Review

**DOI:** 10.3390/life13071587

**Published:** 2023-07-19

**Authors:** Michael F. Dinatolo, Daiana Roxana Pur, Roy Eagleson, Sandrine de Ribaupierre

**Affiliations:** 1 Schulich School of Medicine and Dentistry, Western University, London, ON N6A 5C1, Canada; dpur2024@meds.uwo.ca; 2 Brain and Mind Institute, Western University, London, ON N6A 5B7, Canada; eagleson@uwo.ca (R.E.); sderibau@uwo.ca (S.d.R.); 3 Department of Electrical and Computer Engineering, Western University, London, ON N6A 3K7, Canada; 4 Department of Clinical Neurological Sciences, Schulich School of Medicine and Dentistry, Western University, London, ON N6A 5C1, Canada

**Keywords:** BOLD signal variability, pediatrics, biomarker, fMRI, neurodevelopment

## Abstract

Background: As pediatric BOLD Signal Variability (SV) analysis is relatively novel, there is a need to provide a foundational framework that gives researchers an entry point into engaging with the topic. This begins with clarifying the definition of BOLD signal variability by identifying and categorizing the various metrics utilized to measure BOLD SV. Methods: A systematic review of the literature was conducted. Inclusion criteria were restricted to studies utilizing any metric of BOLD SV and with individuals younger than 18 in the study population. The definition of BOLD SV was any measure of intra-individual variability in the BOLD signal. Five databases were searched: Psychinfo, Healthstar, MEDLINE, Embase, and Scopus. Results: A total of 17 observational studies, including male (n = 1796) and female (n = 1324) pediatric participants were included. Eight studies quantified variability as the amount of deviation from the average BOLD signal, seven used complexity-based metrics, three used correlation measures of variability, and one used the structure of the hemodynamic response function. In this study, 10 methods of quantifying signal variability were identified. Associations and trends in BOLD SV were commonly found with age, factors specific to mental and/or neurological disorders such as attention deficit disorder, epilepsy, psychotic symptoms, and performance on psychological and behavioral tasks. Conclusions: BOLD SV is a potential biomarker of neurodevelopmental and neurological conditions and symptom severity in mental disorders for defined pediatric populations. Studies that establish clinical trends and identify the mechanisms underlying BOLD SV with a low risk of bias are needed before clinical applications can be utilized by physicians.

## 1. Introduction

Variability in the blood-oxygen-level-dependent (BOLD) signal has emerged as a metric with potential clinical relevance. It is no longer viewed as simply “noise” from confounding events during functional magnetic resonance imaging (fMRI) [1,2,3] at its most inclusive, BOLD signal variability, hereafter referred to as BOLD SV, is a measure of the intraindividual change of the measured BOLD signal, a proxy for neural activity. BOLD SV has been associated with age and cognitive function over the lifespan [1], as well as clinical symptoms in eating disorders [2], attention deficit hyperactivity disorder (ADHD) [3], or 22q11.2 deletion syndrome [4]. Although it is true that other physiological pulsations can confound the true neural activation found in the BOLD signal, the predictability of these associations should decrease after regression of these confounding post-analysis techniques such as independent component analysis (ICA) denoising and RETROspective Image CORrection (RETROICOR) [1,5]. This allows for the isolation of neural activation effects from other external and physiological contributions that confound the association and improve the signal-to-noise ratio [1].

Overall, younger individuals are reported to be more variable in neural processing than older populations. Older populations report reduced BOLD SV during aging, primarily in subcortical regions. In addition, correlations have been made between poorer performance and reduced BOLD SD in these regions [6]. Although BOLD SV of individual brain regions presents differing trends across the lifespan, an inverted U-shaped trend of cognitive performance and whole brain variability level is observed over the lifespan. Specific trends in the pediatric period have yet to be investigated [6,7,8,9,10].

Despite the growth of BOLD SV being utilized in pediatric research, there remains inconsistency around its definition and the metrics used to characterize variability. Which metrics exist and which should be used is unclear, rendering BOLD SV challenging to apply or interpret in a standardized manner, especially in a clinical setting. For example, cortical morphology metrics such as cortical thickness confound BOLD SV measurements, but this is not consistently demonstrated or accounted for across all metrics [11].

Understanding pediatric BOLD SV may provide insight into critical neuro-developmental processes including maturation of neurotransmitter systems, pruning and neuroplasticity, myelination and white matter integrity, and functional network changes [7,8,10,12]. For example, higher BOLD SV in medial prefrontal areas comprising the default mode network (DMN) has been shown to positively correlate with ADHD symptom severity [3]. Importantly, infancy and adolescence are unique periods of brain development in which early screening and surveillance can mitigate neurodevelopmental issues. Diagnosing neurological and developmental disorders early on in a child’s life can result in earlier identification, and therefore, improved chances for intervention [2]. BOLD SV has the potential to be established as a neurodevelopmental biomarker and contribute to the diagnosis, prognosis, and treatment of neurological disorders in pediatric development [1].

As pediatric BOLD SV is relatively novel, there is a need to provide a foundational framework that gives researchers an entry point into engaging with the topic. BOLD SV is not yet clearly defined, nor is it measured using a single metric of variability deemed more effective in clinical and research environments. This systematic review attempts to clarify the definition of BOLD SV by identifying and categorizing the various metrics used to measure BOLD SV, and how each metric has been utilized in the literature.

## 2. Materials and Methods

### 2.1. Operational Definitions

BOLD SV, in this review, was defined as any measure of intra-individual variance in the BOLD signal. The BOLD signal was acquired signal correlated with changes to blood flow and blood oxygenation to localized regions of the brain [13]. The signal also had to characterize the flow of oxygenated hemoglobin being used to support neuronal activity [13]. This definition was chosen to ensure a comprehensive set of all definitions, which are presently not well defined in the BOLD SV literature.

### 2.2. Article Search Strategy

The preferred reporting items for a systematic review and meta-analysis (PRISMA) guidelines were used to conduct the systematic review [14]. The electronic literature search was conducted in November 2021 by using MEDLINE (2003 to 2021), Ovid Healthstar (2003–2021), Psychinfo (2003–2021), and Embase (2003 to 2021) through the Ovid platform, as well as the Scopus (2003 to 2021) database. Google Scholar was also searched, although no additional citations were captured. The same strategy was used for each database to search for controlled vocabulary and keywords. These key terms were: BOLD Signal AND (Variability OR standard deviation OR Mean Successive Difference) AND (Paediatric OR Adolescent OR Youth OR Infant). Forward and backward citation tracing was conducted to search for articles that may not have been captured in electronic databases or Google Scholar.

### 2.3. Study Inclusion and Exclusion

Articles included used BOLD SV to measure intra-individual variability, or variability changes across the lifespan. Measures of BOLD SV used to adjust for signal noise and other confounders were excluded. All studies possessed participants in their final study sample that were under the age of 18, even if individuals over the age of 18 were included in the study. The inclusion criteria permitted both studies that used an observational or experimental design. Only results relevant to pediatric samples were included in the final extraction. Review papers, conference abstracts and letters to editor case reports and case studies, non-peer-reviewed studies, populations over 18, non-human studies, non-English papers, editorials, and any study without enough data (i.e., did not identify age) were all excluded.

### 2.4. Study Selection and Quality Assessment

A two-stage screening process was conducted to identify relevant articles. All articles were first identified through electronic database searches and imported into the systematic review management system known as Covidence. Duplicates of captured articles were then removed. Abstract and Title screening was completed by two authors (MD, DRP) who screened inclusively to avoid the removal of potentially relevant articles. Then, a full-text review was independently conducted by two authors (MD, DRP). The review team collaboratively reviewed full-text articles and resolved conflicts. Risk of bias (ROB) and quality assessment was then conducted using the Downs and Black checklist [15].

### 2.5. Data Synthesis

Mean and standard deviations (SD) described the study population’s age and the number of control and diseased patients. In addition, the number and percentage of analysis conducted in studies, and the total number of male and female participants were also calculated from all studies included. Given the heterogeneity of the identified BOLD SV definition subgroups, and of the pediatric populations studied, a meta-analysis was not performed. A data extraction spreadsheet was developed to capture information pertaining to the definitions, study characteristics, sample characteristics, patient characteristics, variability metrics, scales, independent variables, and results of each article. Tables were constructed to summarize study characteristics, BOLD SV metrics and associated findings, and study objectives. Figures were constructed to summarize the results of the search via the PRISMA flowchart, summarize overall findings of Metric type and prevalence, and summarize significant findings in the literature.

## 3. Results

### 3.1. Study Sample

**Figure 1:**
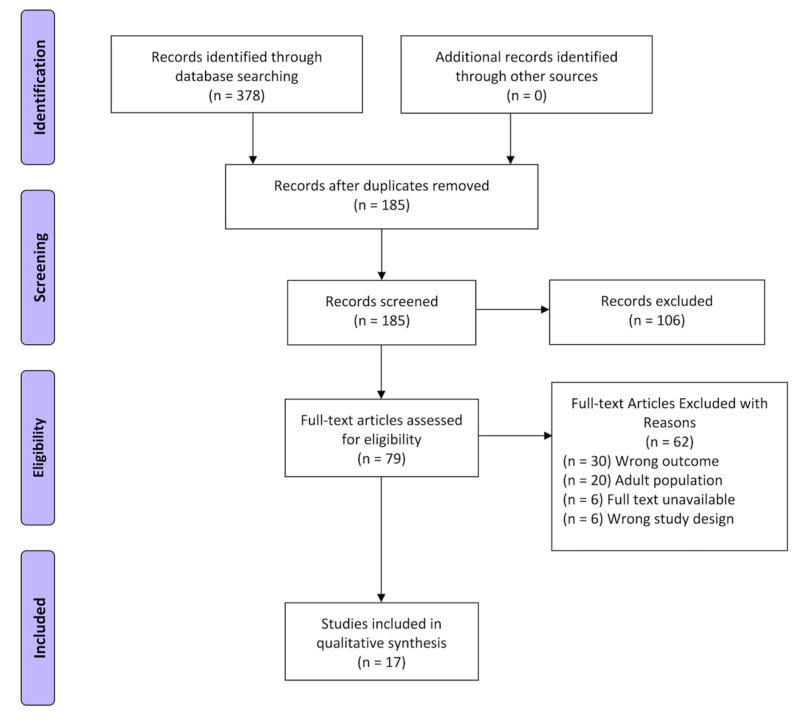
The Preferred Reporting Items for Systematic Reviews and Meta-Analyses (PRISMA) Diagram.

Of the five databases searched, Psychinfo contained 20 results, Healthstar had 41, Medline had 46, Embase had 96, and Scopus had A total of 185 unique studies were identified, and 17 studies were included. The PRISMA flowchart depicts how many studies were included or excluded at each screening step (Figure 1). This study included 10 different metrics of BOLD SV, a total of 3258 participants, and 2869 total pediatrics (1796 M/1324 F). Of the 17 studies, 8 used deviation from the average BOLD signal [4,10,16,17,18,19,20], 4 used correlational measures of BOLD SV [7,21,22,23], 7 used signal complexity [17,19,20,24,25,26,27], and 1 used the structure of the HRF [28] (Table 1). Metrics classification and associated findings can be found in Table 2.

**Table 1:** BOLD Signal Variability Study Characteristics.

Title	Author and Year	Location (Region, Country)	Study Design	Age of Subjects	Sex	Sample Size	Case Definition
Age-Associated Patterns in Gray Matter Volume, Cerebral Perfusion and BOLD Oscillations in Children and Adolescents	Bray et al., 2017 [26]	Calgary, Alberta, Canada	Cross-Sectional	Mean = 13.8, SD = 3.12 Range = 7–18	Typically developing females = 34 Typically developing males = 25	Typically developing = 59	All participants healthy (No cases)
BOLD SV and complexity in children and adolescents with and without autism spectrum disorder	Easson, et al., 2019 [17]	Toronto, Ontario, Canada	Cross-Sectional	ASD Group Mean = 13.25, SD = 2.87 ASD Group Range = [9.6–17.80] Typically Developing Mean = 13.42 SD = 3.21 Typically Developing Range [8.10–17.60]	ASD Males = 20 Typically Developing Males = 17	ASD = 20 Typically Developing = 17 Total Sample Size = 37	Autism spectrum disorder was defined by the Autism Brain Imaging Data Exchange (ABIDE) II database (Where cases ascertained from)
Changes in BOLD variability are linked to the development of variable response inhibition: BOLD variability and variable response inhibition	Thompson et al., 2020 [7]	London, UK	Cross-Sectional	Children Range = [10–12] Children Mean = 11.56, SD = 0.83 Adult Range = [18–26] Adult Mean = 21.55, SD = 2.31	Females = 10 Males = 9	Children 10–12 = 19 Adults 18–26 = 26 Total = 45	All participants healthy (No cases)
Creative internally directed cognition is associated with reduced BOLD variability	Roberts, et al., 2020 [18]	Auckland, New Zealand	Cross-Sectional	Range = [17–25] Mean = 21 years, SD = 4 years	8 Males and 16 Females	24 typically developing	All participants healthy (No cases)
Disentangling resting-state BOLD variability and PCC functional connectivity in 22q11.2 deletion syndrome	Zöller et al., 2017 [4]	Geneva, Switzerland	Case Control	22q11.2 Gene Age Range = [9.0–24.8] Mean 22q11.2 Gene Age = 16.53 ± 4.25 Control Group Age Range = [9.5–24.9] Mean Control Group Age = 16.44 ± 4.20	Males = 21 Females = 29	Healthy Controls = 50 (22/28) 2q11.2DS = 50 (21/29) Total = 100	50 patients with 22q11.2DS, which is a specific type of microdeletion in chromosome 22
Individual Differences in Reading Skill Are Related to Tiral-by-Trial Neural Activation Variability in the Reading Network	Malins et al., 2017 [21]	United States	Cross-Sectional	Discovery Sample Range = [7.8–11.3] Discovery Sample Mean = 9.3, SD = 0.6 Confirmation Sample Range = [7.5–11.3] Confirmation Sample Mean = 9.4, SD = 1.1	Sample 1 females: 18 female Sample 1 males: 26 male Sample 2 females: 14 female Sample 2 males: 18 males	Sample 1 = 44 Sample 2 = 32 Total = 76	All participants healthy (No cases)
Moment-to-Moment BOLD Signal Variability Reflects Regional Changes in Neural Flexibility across the Lifespan	Nomi et al., 2017 [16]	Miami, Florida USA	Cross-Sectional	Slow repetition time Range = [6–85] Slow repetition time Mean = 42.26, SD = 23.60 Fast repetition time Range = [6–85] Fast repetition time Mean = 42.46, SD = 23.30	191 participants, 132 Female, 59 male	191 Participants	All participants healthy (No cases)
Neural correlates of inhibitory control and functional genetic variation in the dopamine D4 receptor gene	Mulligan et al., 2014 [23]	Alberta, Canada	Cross-Sectional	All Participants are 18	Female population = 33 Male population = 29	7R+ = 23 7R− control = 39 Total = 62	(R7+) group (dopamine D4 receptor gene (DRD4) with 7 repeats in the Variable Number of Tandem Repeats section (VNTR) of DRD4)
Neural, electrophysiological and anatomical basis of brain-network variability and its characteristic changes in mental disorders	Zhang et al., 2016 [22]	Nanjing, PR, China	Case Control	Total Study Age Range = [8–25] UM Sample Controls = 15.1 +/− 3.7 Autism = 3.6 +/− 2.4 Peking University-PKU Sample Controls = (11.4 +/− 1.9) ADHD = (12.1 +/− 2.0) New York University-NYU Controls = (12.2 +/− 3.1) ADHD = (12.2 +/−13.1)	Autism UM dataset controls = (48/16) Autism UM dataset Autism = (31/7) ADHD PKU dataset controls = (84/59) ADHD PKU dataset ADHD = (89/10) ADHD NYU dataset controls = (54/54) ADHD NYU dataset ADHD = (106/34)	Autism MU dataset controls = 64 Autism MU dataset Autism = 38 ADHD PKU dataset controls = 143 ADHD PKU dataset ADHD = 99 ADHD NYU dataset controls = 108 ADHD NYU dataset ADHD = 140 Total = 592 (we only use a subset of 1180 total in this study due to age exclusions)	Schizophrenia case definition as defined in the Taiwan Dataset 1 (Guo et al., 2014); and the COBRE Dataset 2. Autism case definition as defined by the definition as defined in the New York University-NYU Dataset 3 and University of Melbourne-UM Dataset 4 (which are from ABIDE Consortium) ADHD case definition was defined by the Peking University-PKU Dataset 5; and New York University-NYU Dataset 6 which are included from 1000 participants derived from the Functional Connectome Project
Psychotic symptoms influence the development of anterior cingulate BOLD variability in 22q11.2 deletion syndrome	Zöller et al., 2018 [29]	Geneva Switzerland	Case-Control	Between 10 and 30 years old	PS+ = 28 (12/16) PS− = 29 (14/15) Healthy controls = 69 (30/39)	22q11.2 gene = 57 Healthy Controls = 69 Total = 126	Chromosome 22q11.2 deletion syndrome (22q11DS) is defined as a neurodevelopmental disorder associated with “a broad phenotype of clinical, cognitive, and psychiatric features”. It is a specific type of microdeletion in chromosome 22
Temporal fractal analysis of the rs-BOLD signal identifies brain abnormalities in autism spectrum disorder	Dona et al., 2017 [24]	Austin, Texas, United States	Case-Control	ASD (12.7 ± 2.4 y/o) 55 age-matched (14.1 ± 3.1 y/o) healthy controls	ASD = 46 male and 9 females, Healthy controls = 38 male and 9 females.	ASD = 55 Healthy Control = 55 Total = 110	ASD and age-matched controls. Definition of ASD defined by NITRC database and the ABIDE project
Variability of the hemodynamic response as a function of age and frequency of epileptic discharge in children with epilepsy	Jacobs et al., 2007 [28]	Germany and Montreal Canada	Cross-Sectional	Range = [5 months-18 years] (Mean and SD not calculated)	12 Female, 25 Male	37	Epilepsy, case definition of epilepsy not explicit but EEG-fMRI data were ascertained in children who met the following criteria: (1) An indication for an anatomical scan on the basis of the necessity to investigate a lesion seen on a prior anatomical MRI scan or to diagnose them with epilepsy syndrome and exclude pathological changes. (2) Participants had frequent spikes (N 10 in 20 min) identified on EEG outside the scanner, without occurrence in bursts.
Evaluation of spontaneous regional brain activity in weight-recovered anorexia nervosa	Seidel et al., 2020 [19]	Germany	Case Control Study	Total Study Range = 15.5–29.7 recAN Mean = 22.06, SD = 3.38 HC Mean = 22.05, SD = 3.34	Healthy Control = 65 Female recAN = 65 female	Healthy Control = 65 recAN = 65 Total = 130	Recovered Anorexia Nervosa (Weight Recovered). Defined as recAN subjects had to (1) maintain a body mass index (BMI) (kg/m^2^) > 18.5 (if older than 18 years) or above the 10th age percentile (if younger than 18 years); (2) menstruate; and (3) have not binged, purged, or engaged in restrictive eating patterns during at least 6 months before the study.
Complexity of low-frequency blood oxygen level-dependent fluctuations covaries with local connectivity	Anderson et al., 2013 [20]	N/A	Cross-Sectional	Range = [7–30] Mean = 8.3, SD = 5.6	Male = 590 Female = 429	1019	Not Specified
Fractal Analysis of Brain Blood Oxygenation Level Dependent (BOLD) Signals from Children with Mild Traumatic Brain Injury (mTBI)	Dona et al., 2017 [25]	N/A	Cross-Sectional	mTBI Subjects = 13.4 ± 2.3 Age-matched Healthy Controls = 13.5 ± 2.34	N/A	mTBI = 15 Healthy Control = 56 Total = 71	Case Control
The longitudinal relationship between BOLD signal variability changes and white matter maturation during early childhood	Wang et al., 2021 [10]	Canada and Australia	Cross-Sectional	Range = 1.97–8.0 years Mean age at intake = 4.42 ± 1.27	Females = 43 Males = 40	83	None
Frequency-specific alterations of the resting-state BOLD signals in nocturnal enuresis: an fMRI Study	Zheng et al., 2021 [27]	China	Case Control	Range approx. = [7–12] NE Patients 9.27(± 1.760) Control 9.68(± 1.601)	NE males = 57 NE Females = 14 Control Males = 19 Control Females = 16	Children with nocturnal enuresis (NE) = 129 Healthy controls = 37	Case Control

Abbreviations: BOLD, Blood oxygen level dependent; SV, signal variability; BOLD_SD,_ blood oxygen level dependent standard deviation, GMV, gray matter volume; fALLF fractional amplitude of low-frequency fluctuations; ASD, autism spectrum disorder; DRD4, Dopamine Receptor D4; VNTR, Variable Number of Tandem Repeats; VNTR 7-repeats present, 7R+; No VNTR 7-repeats present, 7R−; ADHD, attention deficit hyperactive disorder; recAN weight-recovered anorexia; acAN, acute anorexia; fMRI, functional magnetic resonance imaging; resting state, rs; recovered anorexia, recAN; MSSD, mean square successive difference; mTBI, mild traumatic brain injury, NE, nocturnal enuresis.

**Table 2:** Classification of BOLD Signal Variability Metrics.

Metric Type	Authors	Variability Metric	Description	Findings and Associations
Deviation from Average BOLD Signal	(Roberts et al., 2020 [18], Zöller et al., 2017 [4], Zöller et al., 2018 [30], Wang et al., 2021 [10], Anderson et al., 2013 [20])	BOLD_SD_	Quantified the deviation of average BOLD signal from the mean signal.	BOLD Signal variability globally increased with age in all metrics (some regions decrease) BOLD SV in dACC did not change over age in PS+ patients and increased in PS−. Variability increased with age in the DMN. Positively correlated with GE in structural networks and negatively correlated with performance in ASD behavioral severity (SRS). Negative associations with indexes of creativity
	(Nomi et al., 2017a [16], Seidel et al., 2020 [19], Amanda K. Easson and McIntosh 2019 [17])	sMSSD	Calculated by subtracting the amplitude of the signal at time point t from time point t + 1, squaring, and then averaging the resulting values from the entire voxel time course.	
Correlational Measures of BOLD Signal Variance	(Zhang et al., 2016 [22])	Temporal Variance	The BOLD time series were segmented into non-overlapping windows, a whole brain signal measure is obtained using Pearson correlation, and a region’s variability is compared to others.	Lower variability of DMN in schizophrenia, and increased variability in Autism/ADHD. Changes in variability were closely related to symptom scores and in the 10% most variable regions. Variability increases with age in the inhibition network. More variability in the network was associated with less variability in behavioral performance. Low variability in the DMN was correlated with high FC. Lower variability in 7R+ when compared to 7R− when participants successfully inhibited a prepotent motor response. Primarily seen in the prefrontal cortex, occipital lobe, and cerebellum.
	(Malins et al., 2018 [21], and Mulligan et al., 2014 [23])	GLM Derived Variance	GLM produced trial β series estimates of the signal which was used to estimate a variance.	
	(Thompson et al., 2021 [7])	Differences of Residuals	The difference in the variability between the two residual models.	
Signal Complexity	(Amanda K. Easson and McIntosh 2019 [17])	Sample Entropy	SE was used in identifying repetitive patterns in a time series. The degree of regularity of these patterns of activation was also observed, with fewer complex signals being more random.	Positive correlations were identified between entropy, GE, and age. Negative correlations with SRS severity scores and FD in social and non-social tasks, ADIR and ADOS. Grey matter rs-BOLD FD in mTBI patients had reduced FD. Power law exponents remained unchanged or decreased with age and are linearly related to ReHo, which covaried across subjects and gray matter regions. Grey matter rs-BOLD FD in mTBI patients had reduced FD. The fALFF increased with age, distinguishing posterior, and anterior regions. Higher fALFF values in recAN patient’s cerebellum and the inferior temporal gyrus compared to controls. The fALFF decreased in the right insula in children with NE.
	(Dona et al., 2017a [24] and Dona et al., 2017b [25])	Fractal Dimension	Measure of the structural complexity of a signal derived from hurst exponents and quantified structural complexity across different predefined time windows.	
	(Anderson et al., 2013 [20])	Power Law Exponents	Power-based index of sinusoidal amplitudes in the BOLD signal. Signal that follows fractal characteristics that were self-similar within and across frequencies over a time series were measured.	
	(Seidal et al., 2020 [19], Zheng et al., 2021 [27], Bray 2017 [26])	Fractional amplitude of low-frequency fluctuations (fALFF)	The ratio of the low-frequency power spectrum, specifically in the range of 0.01–0.08 Hz, to the entire signal frequency range.	
Structure of Hemodynamic Response Function	(Jacobs et al., 2008 [28])	HRF Structure	Using the structure of the HRF, like peak time, amplitude or other signal characteristics not mentioned above.	Could not identify an age-specific HRF. Longer peak times of the HRF 0 to 2 yrs.

Abbreviations: BOLD, Blood oxygen level dependent; BOLD_SD,_ blood oxygen level dependent standard deviation; fALLF fractional amplitude of low-frequency fluctuations; ASD, autism spectrum disorder; ADHD, attention deficit hyperactive disorder; FD, fractal dimension; HRF, hemodynamic response function; HRF, hemodynamic response function; recAN weight-recovered anorexia; acAN, acute anorexia; ReHo, regional homogeneity fMRI, functional magnetic resonance imaging; resting state, rs; recovered anorexia, recAN; MSSD, mean square successive difference; mTBI, mild traumatic brain injury. A total of eight studies measured variability as a deviation from the mean BOLD signal time series [4,10,16,17,18,19,20,30]. Of these, five studies utilized BOLD_SD_ as a measure of variability [4,10,18,20,30], and three used mean sample standard deviation (MSSD) [16,17,19] of the BOLD signal. In addition, four studies utilized correlational metrics to quantify the variability in the BOLD Signal [7,21,22,23]. This included two articles producing a beta series derived from linear modeling or regression to quantify variability [21,23], and one with a difference of residuals-based calculation [7]. Seven studies were identified that used a complexity-based metric of BOLD SV [17,19,20,24,25,26,27]. Of these, three used Fractional amplitude of low-frequency fluctuation (fALFF) as a metric of variability [19,26,27], two used fractal density (FD) [24,25], one used sample entropy (SE) [17], and one used power law exponents [20]. Finally, only one study used changes in key features of the hemodynamic response as a measure of variability [28]. Figure 2 is a graphical representation of the four categories of variability metrics.

**Figure 2:**
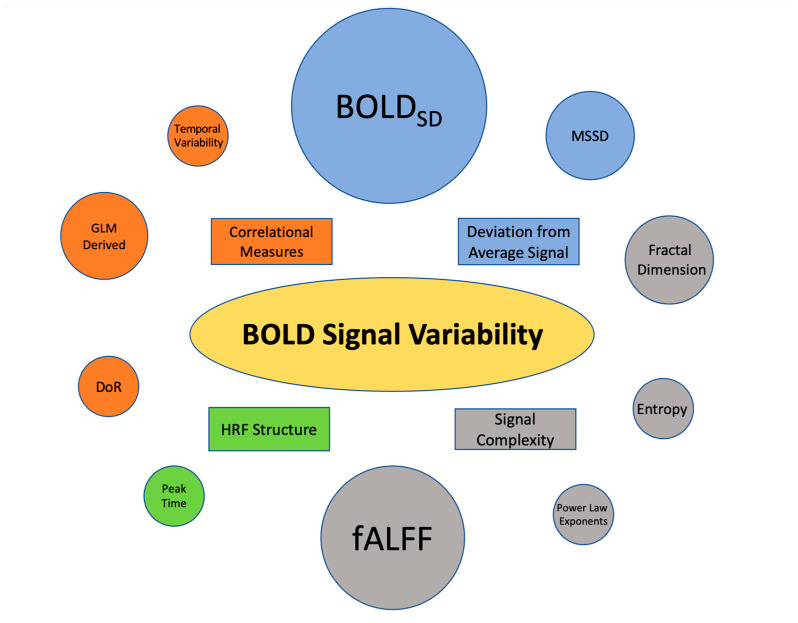
Summary of BOLD SV Metric Prevalence in the Pediatric Literature. Circles of the same color belong to a common metric subtype. The area of each circle is proportionate to the number of papers published that utilized the metric. BOLDSD appeared in six papers, making the largest circle. Colors were used to indicate metrics belonging to a common subtype of variability (i.e., Deviation from Average BOLD Signal). Abbreviations: BOLD_SD_, Blood Oxygen Level Dependent Signal Standard Deviation; MSSD, Mean Successive Square Difference; fALFF, Fractional amplitude of low-frequency fluctuation; HRF, Hemodynamic Response Function; DoR, Difference of Residuals; GLM, General linear Model.

### 3.2. Study Characteristics

As presented in Table 1, all studies were observational. Of these 11/17, (65%) are cross-sectional studies and 6/17 (35%) are case control. Here, 11/17 of the studies exclusively included children (those under the age of 18) and 6/17 included a mixed population that included more than one child in their study population.

The statistical analyses performed in the included studies were heterogenous, with the most common being partial least squares analysis (n = 4, 24%) [10,17,18,30], followed by temporal fractal analysis (n = 2, 12%) [24,25] and principle component analysis (PCA) (n = 1, 5.9%) [26]. Age-based variability trends (n = 5, 29.4%) [4,16,26,28,30], behavioral and psychological task performance (n = 2, 12%) [18,21], and mental disorders (n = 5, 29%) [17,19,22,24,30], neurological disorders (e.g., Autism, ADHD, schizophrenia/psychotic symptoms, and anorexia) (n = 3, 18%) [25,27,28], and genetic conditions (n = 3, 18%) [4,23,30], were examined in relation to BOLD SV. Table A1 indicates the various objectives of the included articles.

### 3.3. BOLD SV Metrics

Variability metrics were grouped into four categories: BOLD signal deviation from the mean, measures of BOLD SV derived through a correlational analysis of the signal, measures of BOLD signal complexity, and measures that utilize characteristics of the hemodynamic response function (HRF). Table 2 presents the descriptions of each variability metric identified in the review.

### 3.4. Findings Associated with Deviation from the Average BOLD Signal

#### 3.4.1. Standard Deviation of the BOLD Signal (BOLD_SD_)

Five studies utilized BOLD_SD_ (n = 5, 72.5%) [4,10,18,20,30] quantifying an average amount of deviation from the typical BOLD signal (Table 2). Most studies had the objective of characterizing variability differences between healthy or typically developing controls and those with a mental disorder, neurological issue, or genetic condition (n = 2, 40%) [4,30]. The next most common objective was identifying lifespan and age-based patterns in variability (n = 2, 40%) [4,30]. Only one of these studies looked at structural changes across the lifespan (n = 1, 20%) [8], while the rest looked at network-based changes (n = 4, 80%) [4,10,20,30].

A longitudinal study of white matter structure in healthy children, and a cross-sectional study on 22q11.2 deletion syndrome, assessed aging and its effect on BOLD_SD_ of the brain and age-related variability patterns, respectively. A global association of increasing BOLD_SD_ with age, particularly in the frontal gyrus, supramarginal gyrus, middle temporal gyrus, and superior parietal lobule was reported [10]. BOLD SV and white matter micro and macro structure metrics such as white matter volume, mean fractional anisotropy (FA), and mean diffusivity (MD) measured at younger ages were predictive of BOLD SV at older ages [10]. BOLD_SD_ associations with these macro and micro structural alterations changed over the lifespan and across various regions throughout the brain.

Two studies focused on 22q11.2 deletion syndrome, a genetic disorder commonly associated with schizophrenia, and its relationship to BOLD_SD_ [4,30]. Strong positive psychotic symptoms (PS+) were associated with aberrant age relationships and concurrently saw BOLD SV increase in visual regions and decrease in the cortices of the prefrontal and orbitofrontal regions of the brain [4]. Both of the studies identified elevated and reduced BOLD_SD_ across different brain regions, often being lower in regions of the DMN (medial prefrontal cortex, posterior cingulate cortex (PCC), and lateral parietal cortex) [4,30]. Notably, the lack of association between age and BOLD_SD_ was identified in the dACC or DMN of patients with high psychotic symptom scores (PS+) [30] or schizophrenia, which was typical of healthy controls [4,30] and populations with less severe psychotic symptoms (PS-) and the 22q11.2 deletion [30]. This resulted in globally reduced variability in the dACC region of children with PS+ when compared to children with PS- [30].

A task-based study measured internally directed creative cognition using a future simulation task, and an alternate uses task (AUT) also has correlations with BOLD_SD_. Performance, which acts as an index of creativity, was negatively correlated with the BOLD_SD_ [18].

#### 3.4.2. Mean Successive Squared Difference (MSSD)

MSSD is another way deviation from the average signal is measured (Table 1). Three studies utilized MSSD as a metric of BOLD SV [16,17,19] (n = 3, 27.5%)**.** Although heterogeneous, studies in this section focused on diseased-based findings, one study assessed BOLD SV’s relationship to autism spectrum disorder (ASD) [17] and another assessed lifespan-related trends in various networks across the brain [16]. The third study looked at recovered anorexia patients but found no significant findings using the metric [19].

A cross-sectional study of ASD and typically developing individuals used MSSD as a metric to quantify BOLD SV. Variability increased linearly in the SN nodes (anterior insula) and the ventral temporal cortex and decrease across subcortical, visual, sensorimotor, DMN, and central executive network (CEN) regions [16,17]. When MSSD was used in a population of children with ASD, positive associations between MSSD of the BOLD time series and GE in structural networks are present. Brain regions that had positive correlations with GE also had a negative correlation with behavioral severity scores such as the social responsiveness scale (SRS) [17].

### 3.5. Findings Associated with Correlational Measures of BOLD SV

The following section includes all metrics that attained variability measures through correlation-based methods (n = 4, 24%). This includes temporal variability, GLM-derived measures of variability, and the BOLD% signal change. Appendix A Table A2 includes the definitions of these metrics.

#### 3.5.1. Temporal Variability

Temporal variability was assessed in one study, which included subjects with mental disorders and healthy controls (n = 1, 25%) [22]. In typically developing children, age-related trends demonstrated significant increases in temporal variability across the inhibition network, from childhood to adulthood [22]. When characterizing relationships in mental disorders, children with schizophrenia had decreased BOLD SV in DMN regions associated with higher activity and connectivity compared to typically developing patients. This decreased variability was also associated with neurocognitive symptoms characteristic of schizophrenia [22]. Increased variability was seen in subcortical regions (thalamus, putamen, and pallidum) in these patients, while children with ADHD, saw increased BOLD SV in regions of the DSN and decreased BOLD SV in subcortical regions. Variability levels were not the same in the DMN regions of typically developing children with autism and ADHD, with the medial frontal areas mainly affected in ASD, and the posterior cingulate in ADHD [22]. Importantly, regions with the highest variability in controls, (i.e., trans modal areas) have lower levels of variability in disorders [22]. Those areas in controls with the lowest variability, such as primary sensory regions, are more prevalent in mental disorders [22].

According to a study, 50% of regions with significant changes in BOLD SV in the three disorders are in the top 10% of regions with the highest or lowest variability in controls [22]. Negative correlations between the variability of the signal in a brain region and its level of activity were found. Low DMN variability was consistently identified alongside strong functional connectivity (FC) within the DMN during resting state fMRI [22].

#### 3.5.2. Multilinear and General Linear Model (GLM)-Derived Variance Measurement

Two studies used multilinear models or GLM-derived variance measures (n = 2, 50%) [21,23]. A reading skill task was used to investigate deviations from the mean BOLD signal by measuring the standard deviation of a beta series representing mean activation. In the left inferior frontal gyrus pars triangularis, the SD of the series appears to account for additional variance in reading skill, measured as task performance [21].

Only one study measured BOLD SV as the % change in signal. This study was task-based, studying a “Go/No-Go” behavioral task in children and assessed neural factors associated with inhibitory control and genetic variation in the Dopamine (DA) receptor gene, having seven repeats in the variable number of tandem repeats (VNTR) of the DA receptor gene DRD4 (7R+). The presence of seven repeats in the VNTR region of DRD4 (7R+) is associated with psychiatric disorders that present self-regulation issues such as ADHD [23]. Lower variability was found in those with 7R+ when compared to 7R− groups during successfully inhibited prepotent motor response. This was observed in two regions located in the prefrontal cortex, one in the cerebellum and one in the occipital lobe [23]. There were no differences in behavioral performance of the “Go/No-Go” task and correlations between task-related BOLD responses were not observed during the task [23].

#### 3.5.3. Difference of Residuals

One cross-sectional study used the difference of residuals metric (n = 1, 25%) [7]. This variability metric compares the difference in variability between the two residual models of observed and expected BOLD response. BOLD SV in the inhibition network was reported as lower in children than adults during a successful stopping task [7].

### 3.6. Findings Associated with Signal Complexity

Signal complexity (as defined in Table 2) was also used to measure the variability in the BOLD signal. Signal complexity is also described as the unpredictability of a signal over its time series [31]. Seven studies were identified that utilized this metric (n = 7, 41%) [17,19,20,24,25,26,27].

#### 3.6.1. Entropy/Sample Entropy

Another way BOLD SV is estimated is by using an entropy metric such as sample entropy (SE). SE is used to identify repetitive patterns in a time series, and the degree of regularity of patterns of activation observed [31].

Only one study used an entropy-based metric of BOLD SV in populations of children with and without autism spectrum disorder (n = 1, 14%) [17]. Distributed brain regions showed increases in MSSD and entropy from childhood through adolescence and positive correlations between entropy, general efficiency (GE), and age in both ASD and typically developing groups [17]. Negative correlations with SRS scores and entropy [17]. Lower levels of sample entropy are seen in ASD individuals during social and non-social tasks [17].

#### 3.6.2. Fractal Dimensionality

Two studies used fractal dimension, obtained by fractal analysis, as a measure of the complexity derived from hurst exponents (n = 2, 29%) [24,25]. FD is a statistical measure of how completely a fractal appears to fill the space in the geometric sense. When used for signals, it can become a metric of structural complexity across a given time domain [32].

Reduced signal complexity was seen in ASD participants with respect to controls in the amygdala, the vermis, the basal ganglia, and the hippocampus. Decreases were correlated with autism diagnostic interview-revised (ADI-R) and autism diagnostic observation schedule (ADOS) scores [24]. The nucleus accumbens and the caudate head showed significantly reduced fractal dimension. Regions of the cerebellum in the ASD cohort showed significantly reduced FD, particularly in the vermis with mild correlations with the Autism Diagnostic Interview restricted and repetitive behaviors (ADIRRB) and Autism Diagnostic Observation Schedule Restricted and Repetitive Behaviors (ADOSRRB) metrics [24].

A cross-sectional study of children with mTBI utilized FD as a metric of BOLD SV. There were 11 brain regions where FD significantly decreased for mTBI patients, including the caudate nucleus and nucleus accumbens [25]. The FD also decreased for mTBI patients when compared with the uninjured control group in both these areas [11].

#### 3.6.3. Power-Based Metrics

Power or spectral density-based variability metrics are an index of the signal amplitude of sinusoidal oscillations within and across frequencies over a time series [8]. This signal, which demonstrates scale-free behavior, requires fractal-like self-similarity in a spatial or temporal scale to use a power law measure of complexity [20].

Only one study used this metric to quantify complexity in healthy participants (n = 1, 14%) [20]. Increases in complexity were found throughout the whole brain during adolescence and early adulthood, excluding the DMN and attention control networks. Complexity did not change with age in a subset of gray matter regions and dorsal attention networks [20]. Decreases in complexity were observed in other regions of the brain, but the largest reductions occurred in the subcortical gray nuclei [20]. A strong positive correlation between local connectivity (ReHo) and complexity in endogenous brain activity fluctuations was also identified [20]. White matter and areas of gray matter with lower local connectivity exhibited more randomness in their BOLD fluctuations. In the basal ganglia, thalami, and spinocerebellum. relatively lower complexity than would be predicted from ReHo [20] was observed.

#### 3.6.4. Fractional Amplitude of Low-Frequency Fluctuation (fALFF)

Three studies utilized fALFF as a metric of complexity (n = 3, 43%) [19,26,27]. One cross-sectional study found that fALFF metrics were associated with age, with 5.2% of the variability in age attributed to complexity. They distinguished areas of the DMN and salience network in occipital, temporal, superior parietal, and pre- and post-central gyral regions. The age-associated fALFF component was also distinguishable from the posterior from anterior cortical regions. Anterior regions of the DMN had a more evident decline compared to posterior regions not a part of the DMN [26].

Two studies identified used fALFF in populations with mental disorders or neurological conditions [19,27]. It was found that in children with nocturnal enuresis (NE), fALFF was higher in the right insula and in the typical spectral band. Regional Homogeneity (ReHo) rose in the left insula and the right thalamus in children with NE, and the right insula saw increased fALFF in NE patients [27]. In the slow-5 frequency band, fALFF increased in the superior cerebellum and superior temporal gyrus in those with NE. The fALFF in slow-2 was primarily seen in white matter and was observed to be negative in other bands [27]. In anorexia nervosa patients and healthy controls, values indicated alterations in the temporal gyrus and cerebellum of recovered anorexic patients. Between-group differences in fALFF were also observed in the cerebellum, specifically in the vermis [19].

### 3.7. Findings Associated with Characteristics of the Hemodynamic Response Function (HRF)

The hemodynamic response function (HRF) describes the behavior of the BOLD response over time by measuring the change in the HRF with respect to time. A single cross-sectional study uses the “time to peak” in the function, as well as the overall shape, as a metric of variability.

A study of pediatric epilepsy patients discovered that the shape of the HRF changes in children, specifically, the amplitude decreases significantly with greater EEG spike frequency in epileptic patients. When looking at age-related trends in these patients, the intrasubject variability of the amplitude of the HRF does not vary significantly across the age groups of epileptic children [28]. In epilepsy cases, authors reported differences in age could not be distinguished using time-to-peak or amplitude-based metrics of the HRF [28].

## 4. Discussion

### 4.1. Summary of Evidence

#### 4.1.1. Metric Utilization

A total of 17 studies and 10 unique metrics of BOLD SV were included in this review. These metrics were categorized into four types of variability measures: Eight used deviation from average BOLD signal, four used correlational measures of BOLD SV, seven used signal complexity, and one used the structure of the HRF. In addition, only seven studies included healthy controls (HCs) exclusively, while 10 included patients with neurological, psychiatric, or genetic disorders.

Presently, deviation-based and complexity-based metrics appear to be the most viable for clinical application and utilization in pediatric BOLD SV research, as the majority of studies included that used these metrics, produced significant results. In addition, complexity and deviation-based methods of quantifying signal variability have been previously established in other neuroscientific contexts that use biomedical signal analysis such as brain variability, heart rate variability, and variability in respiratory rates [33,34]. This attests to these metrics’ promise as future biomarkers of BOLD SV. These metrics have been associated with neurophysiological, psychological, genetic, and developmental findings and have been presented in these studies. The pediatric BOLD SV literature specifically includes global and regional changes or associations of variability and neurostructural alterations, factors relating to mental and neurological disorders, genetic markers of disease, and psychological performance and aging (Table 2).

Deviation-based metrics can be used in the BOLD SV literature to describe how much the magnitude of the BOLD signal changes in the *y*-axis compared to the baseline level of activation over the course of the time series. Deviation-based metrics appear to be more versatile, as they are used to assess pathological or non-pathologically related trends in neuropsychological development [4,18]. This includes developmental, pathological, and psychologically relevant findings in the BOLD SV literature (Table 2). Complexity metrics best describe the degree of structure and information of signals and how associated they are with other functional networks [35]. Signals can have equal amounts of deviation from the average but still differ in complexity. Studies included in this review report on only developmental or age-related trends, neurological pathologies, and mental disorders (Table 2) since aging and pathologies of the nervous system result in signal degradation over time [35,36,37]. Typically, the more complex a signal is, the less it is impacted by unhealthy pathologies or age-related complexity degradation, and the metric should be utilized with this in mind.

Non-deviation or non-complexity-based subgroups include HRF metrics and correlational BOLD SV metrics. HRF-based variation currently is not well established or justified as an effective BOLD SV metric, given the single study using the HRF variability metric did not show significant results [28]. Correlational metrics are highly specific to statistical models utilized in the study, and it is unclear how useful they will be unless they can be applied to clinical settings in a standardized manner. Unlike the HRF, however, they have produced significant results that have established pediatric BOLD SV trends [21,22].

#### 4.1.2. The Inverted U Trend and BOLD SV

Global increases in whole brain BOLD SV were associated with aging in three of the four metric types in pediatric populations. The literature has competing perspectives on age-based variability trends. Typically, an inverted U-like pattern of variability is reported over the lifespan, suggesting that variability is functionally related to cognitive performance, and increases through childhood to adulthood and decreases in older age [8]. Multiple studies included in this review demonstrate that in both resting state and task-based protocols, whole brain variability increased from 0–18 years of age [4,17,24,30]. This inverted U may relate to the development of cognitive capacity, dynamic range, and therefore, efficiency that increases in childhood, peaks in young adulthood, and declines in older age [8]. One cross-sectional study that was identified proposed that lifespan-based trends in BOLD SV are better characterized by networks that simultaneously increase and decrease in variance over the lifespan. They report resting state fMRI data, which indicates regions of the SN nodes (anterior insula) and the ventral temporal cortex increase while there are decreases across subcortical, visual, sensorimotor, DMN, and CEN regions [16].

Though not established yet in pediatrics, the inverted U trends of cognitive performance and variability over the lifespan also follow dopamine signaling strength [38]. Cognition appears to change as DA signaling strength becomes too low or too high, and may play a role in the change in variability throughout the lifespan [38,39]. It should be noted that higher levels of BOLD SV have been specifically associated with elevated levels of cognitive flexibility [8]. In younger and older adults, suboptimal dopamine synthesis capacity is also associated with reduced cognitive flexibility, in addition to reduced BOLD SV [38,40].

#### 4.1.3. BOLD SV Trends in Mental and Neurological Conditions

BOLD SV trends were identified in individuals with ASD, ADHD, schizophrenia, epilepsy, NE, recovered anorexia mTBI, and VNTR 7R deletion syndrome. In all disorders, atypical variability trends were uniquely observed across a variety of brain regions and compared to health controls. Regions associated with mental disorders, brain injury, and higher symptom severity were also associated with changes in variability.

**Figure 3:**
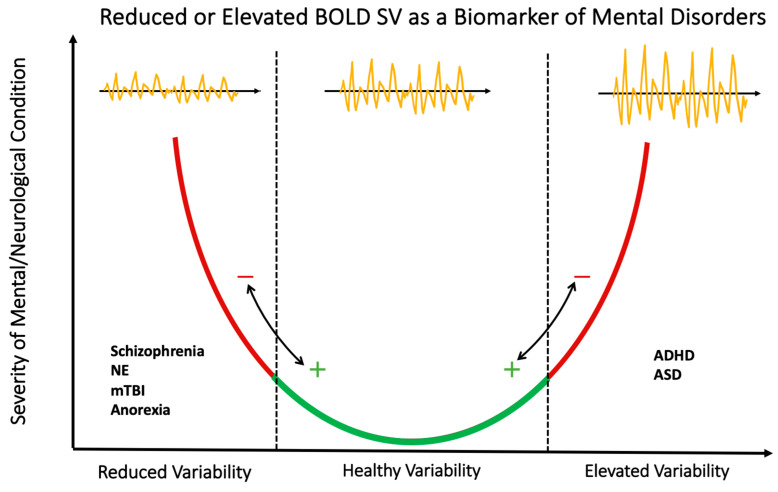
Reduced or Elevated BOLD SV as a Biomarker of Mental Disorders. When symptom severity scores and other markers of mental and neurological condition severity are present, BOLD SV is often reported as elevated or reduced in regions associated with the condition. Though it has promise as a biomarker, this model is only a representation of trends in the present literature. Abbreviations: NE, nocturnal enuresis; mTBI, mild traumatic brain injury, ADHD, Attention Deficit Hyperactive Disorder; ASD, autism spectrum disorder.

Interestingly, lower variability of DMN specifically was seen in those with schizophrenia, while increased DMN variability in Autism/ADHD was identified, reporting opposite relationships [22]. Schizophrenia and ASD/ADHD’s opposite variability trends in similar regions may be a result of their differing function in the social development patterns associated with the two diseases [3,29,41]. In the identified mental disorders, variability trends were inconsistent across regions, with some showing increases, aligning with the global variability trend, while others showed decreases or no change, breaking the trend. Elevated and reduced variability levels compared to healthy controls were consistent across all studies with a focus on mental disorders and neurological conditions, often in affected regions (Figure 3).

### 4.2. Recommendations for Clinical Applications

Though it is likely that deviation-based or complexity-based metrics have the potential to be used as a clinical biomarker in the future, not enough information is known at this time to make specific assessments on which metrics if any should be used and applied to clinical environments at this stage. More studies conducted using these metrics will allow for a more comprehensive appraisal of BOLD SV metrics. For instance, each metric’s ability to be used in combination with signal processing and statistical techniques to mitigate confounding from non-neuronal sources of BOLD SV should be considered before clinical utilization.

Although the variability in neuronal activity is a key component of BOLD SV, there are other significant contributing sources to variability before processing, filtering, and statistical modeling, and ICA techniques are applied to the signal. Non-neuronal physiological sources of BOLD SV include brain hemodynamics influenced by heartbeat, respiration, and low-frequency oscillations (LFOs). LFOs in and of themselves are derived from a multitude of sources, potentially including Mayer waves, vasomotion from oscillations in vascular tone, CO_2_ vasodilation, variations in heart rate or respiratory volumes, gastric oscillations seen using electrogastrograms, and aliased signals of cardiac and respiration due to long signal repetition times (TRs) [42].

These three categories of physiological factors contribute to BOLD SV due to their ability to impact fluctuations in oxygenated hemoglobin concentration in various regions of the brain. Heart rate, vasodilation vasoconstriction, respiratory rate, and respiratory depth over the course of the signal can all cause oxygenated hemoglobin concentration to change independent of brain region activation. This can result in the true associations or effects of BOLD SV being confounded by non-neuronal sources [43]. For instance, age or disease-related correlations with decreased BOLD SV could truly be a result of a decrease in resting cerebral blood flow, cerebral metabolic rate of O_2_ consumption, and vascular reactivity from aging or disease-related causes. If this variability is not removed or controlled for in the analysis inferences from study conclusions cannot be validly applied to clinical practice [43,44].

These non-neuronal sources of signal can contribute between 20% and 70% of total BOLD SV prior to filtering, component analysis, or other correction steps [42]. Non-neuronal LFO variability is a particularly important target for controlling confounding. The LFO frequency range is where the neuronally related contributions to the BOLD signal can be found and is generally within the range of the low-frequency band (0–0.15 Hz) [45,46]. This is a consequence of the speed at which neuronal activation occurs relative to confounding physiological sources of BOLD SV [45,46]. Caution should be exercised when preparing raw signal for analysis. Approximately 30% of BOLD SV in grey matter is non-neuronal BOLD SV in the same 0–15 Hz frequency range that neuronal BOLD SV is found [42,45,46]. Each contributor to non-neuronal BOLD SV must be mitigated or removed from the signal through statistical modeling to control for these sources of variability. This should be carried out post spectral filtering of the BOLD signal, which already removes 10–15% of the variability in the BOLD signal derived from respiration and cardiac factors [43,47]. If certain metrics are better equipped to isolate neuronal BOLD SV in this frequency range, they should be favored in clinical and non-clinical practice.

Future areas of investigation into BOLD SV should continue to focus on developing BOLD SV into a biomarker of neurodevelopment and a risk factor for neurological issues and mental disorders such as schizophrenia would be a critical advancement of the pediatric BOLD SV literature. Reduced or elevated levels of BOLD SV in particular may identify the need for early intervention or treatment in pediatric populations [48]. In addition, given global variability’s associations with age in childhood development, there are benefits to producing standardized thresholds of BOLD SV in a typical healthy patient across the different neurodevelopmental milestones in different populations (healthy, diseased, injured, etc.). This can be for associated brain regions, networks, or across the whole brain. This would allow for the establishment of BOLD SV as a biomarker of neurodevelopment and neurological conditions. To achieve this, consistent use of BOLD SV metrics in the literature and the recruitment of larger pediatric cohorts with a low risk of bias is vital. For BOLD SV to be a useful clinical biomarker, researchers must produce normative and non-normative distributions of BOLD SV for various regions of the brain in healthy populations and those affected by different neurodevelopmental disorders, respectively.

### 4.3. Future Directions and Limitations

Given the plethora of metrics identified, future studies should seek to implement multiple variability metrics into their analysis to validate that their findings are consistent, while simultaneously ensuring high-quality evidence is procured. Although it appears deviation-based and complexity-based metrics are most utilized, complexity-based metrics were primarily utilized in assessing associations between BOLD SV and either aging or developmental trends, or neurological pathologies or mental disorders. To verify if these metrics are more effective at characterizing variability in these populations, more work must be done to logically standardize these metrics in a way that highlights the strengths and weaknesses of each from a signal-processing perspective. Each metric should be utilized across diverse populations of patients so that findings may contribute to a future common framework for BOLD SV metric utilization.

A complimentary risk of bias analysis was conducted (Table A2), identifying that included articles displayed a risk of bias for external and internal validity overall. Using the Modified Downs and Black checklist identified total scores of 64% for reporting bias, 37% for external validity, 42% for internal validity bias, 37% for internal validity confounding, and an 11% score for power (Table A2). An overall score of 47% was obtained from all articles (Table A2). Criteria that were not included in studies were given a score of 0 unless otherwise indicated. In addition, seven studies included healthy controls while the rest were cross-sectional and included none. Of the 17 studies, 10 (n = 10, 59%) studies included patients with neurological, psychiatric, or genetic disorders. Many of the findings identified come from cross-sectional and case-control studies with a risk of bias. These studies are both observational and non-randomized, making it difficult to make etiologic or casual statements regarding risk factors’ effect on variability. Higher quality evidence with a lower risk of bias will be important to the future of this promising and developing field in order to validate present trends in the literature [38,39]. Once studies on BOLD SV present higher-quality results in terms of typical and atypical pediatric populations, BOLD SV can be used as an important biomarker for neurodevelopment.

## Data Availability

No new data were created or analyzed in this study. Data sharing is not applicable to this article.

## Appendix A

**Table A1:** Objectives of Included Studies.

Author and Year	Title	Objectives of Included Articles	Application
Bray et al., 2017 [26]	Age-Associated Patterns in Gray Matter Volume, Cerebral Perfusion and BOLD Oscillations in Children and Adolescents	Characterize patterns in GMV, CBF, fALFF, age, and their relation to structural and functional connectivity.	Identification of structural and functional relationships in pediatric brain development
Easson, et al., 2019 [17]	BOLD Signal Variability and complexity in children and adolescents with and without autism spectrum disorder	Assess both BOLD variability and BOLD complexity in children and adolescents with ASD and age-matched typically developing participants.	Identification of pathophysiology of ASD
Thompson et al., 2021 [7]	Changes in BOLD variability are linked to the development of variable response inhibition: BOLD variability and variable response inhibition	Effect on intra-individual variability in a response inhibition task and behavioral variability across the lifespan.	Identification of response inhibition trends during development
Roberts et al., 2020 [18]	Creative internally directed cognition is associated with reduced BOLD variability	Identify the relationship between BOLD variability, mean BOLD Signal, and creativity tasks that required flexible thinking in various fMRI tasks.	Identify trends of BOLD SV and creativity/flexibility
Zöller et al., 2017 [4]	Disentangling resting-state BOLD variability and PCC functional connectivity in 22q11.2 deletion syndrome	Investigate relationships between 22q11.2 deletion syndrome associated alterations in BOLD SV and age-relationship throughout the cortex of children and adolescents.	Clinical: identify pathophysiology and symptom severity of 22q11.2 deletion and schizophrenia
Malins et al., 2018 [21]	Individual Differences in Reading Skill Are Related to Tiral-by-Trial Neural Activation Variability in the Reading Network	Determine whether trial-by-trial neural activation variability accounts for variance in reading skill and in what direction.	Identify trends of BOLD SV and variance in reading skill
Nomi et al., 2017 [16]	Moment-to-Moment BOLD Signal Variability Reflects Regional Changes in Neural Flexibility across the Lifespan	Determine regional variability trends across the lifespan using rs-fMRI data across ages 6–85.	Identify brain variability trends in healthy brain development
Mulligan et al., 2014 [23]	Neural correlates of inhibitory control and functional genetic variation in the dopamine D4 receptor gene	Demonstrate carriers of DRD4 (7R+) have reduced prefrontal inhibitory control than non-carriers (7R−) confirmed by locally reduced BOLD % signal change and Go/No-Go task performance.	Clinical: identify pathophysiology of genetic alterations (addition of 7 tandem repeats) of the DRD4 receptor gene
Zhang et al., 2016 [22]	Neural, electrophysiological, and anatomical basis of brain-network variability and its characteristic changes in mental disorders	Characterize temporal variability of the functional architecture of pediatrics with mental disorders in associated regions and networks and establish functional-structural relationships.	Clinical: identify pathophysiology and symptom severity of ADHD, ASD, and schizophrenia and identify SV trends in typically developing patients
Zöller et al., 2017 [29]	Psychotic symptoms influence the development of anterior cingulate BOLD variability in 22q11.2 deletion syndrome	Use the BOLD signal and BOLD_SD_ to assess brain dynamics in patients with psychotic symptoms seen in schizophrenia.	Clinical: identify pathophysiology and symptom severity of schizophrenia
Dona et al., 2017 [24]	Temporal fractal analysis of the rs-BOLD signal identifies brain abnormalities in autism spectrum disorder	Utilize a model-free complexity analysis based on the fractal dimension of the rs-BOLD signal to identify BOLD SV trends in children with ASD.	Identify pathophysiology and symptom severity ASD
Jacobs et al., 2008 [28]	Variability of the hemodynamic response as a function of age and frequency of epileptic discharge in children with epilepsy	Identify age-related changes in the HRF after interictal epileptic discharge and identify the relationship between the number of spikes and a change in the HRF.	Clinical: Identify Pathophysiology of Epilepsy/interictal epileptic discharge in development
Seidel et al., 2020 [19]	Evaluation of spontaneous regional brain activity in weight-recovered anorexia nervosa	Investigate intrinsic regional brain activity differences between recAN, acAN, and healthy controls using resting-state measures of fALFF, ReHo (as reported in acAN, MSSD, DC, and VHMC (as new measures of spontaneous regional brain activity).	Clinical: Identify long-term effects and pathophysiology of recAN and patients
Anderson et al., 2013 [20]	Complexity of low-frequency blood oxygen level-dependent fluctuations covaries with local connectivity	Determine if BOLD fluctuations exhibit temporal complexity associated with local connectivity (ReHo), across gray matter regions utilizing power law behavior.	Identify associations between grey matter ReHo and temporal complexity
Dona et al., 2017 [25]	Fractal Analysis of Brain Blood Oxygenation Level Dependent (BOLD) Signals from Children with Mild Traumatic Brain Injury (mTBI)	Utilize FD to quantify local neural activity in the brain as a metric of functional changes in mTBI patients.	Clinical: Identification of pathophysiology of mTBIs.
Wang et al., 2021 [10]	The longitudinal relationship between BOLD signal variability changes and white matter maturation during early childhood	Investigate longitudinal relationships between BOLD SV, age, and white matter structure in early childhood.	Identify variability trends between brain region SV, structure, and age.
Zheng et al., 2021 [27]	Frequency-specific alterations of the resting-state BOLD signals in nocturnal enuresis: an fMRI Study	Use associations of spontaneous brain activity and scores on urinary intention-related wakefulness in 5 frequency bands to detect abnormalities of activity in local brain regions of children with nocturnal enuresis.	Identify pathophysiology of NE

Note on Abbreviations: BOLD, Blood oxygen level dependent; SV, signal variability; BOLD_SD,_ blood oxygen level dependent standard deviation, GMV, gray matter volume; CBF, cerebral blood flow; fALLF fractional amplitude of low-frequency fluctuations; ASD, autism spectrum disorder; DRD4, Dopamine Receptor D4; ADHD, attention deficit hyperactive disorder; FD, fractal dimension; HRF, hemodynamic response function; recAN weight-recovered anorexia; acAN, acute anorexia; ReHo, regional homogeneity fMRI, functional magnetic resonance imaging; resting state, rs; recovered anorexia, recAN; MSSD, mean square successive difference; DC, degree centrality; VHMC, inter-hemispheric synchronicity; mTBI, mild traumatic brain injury, NE, nocturnal enuresis.

**Table A2:** Scores from the Downs and Black Checklist for Quality Assessment.

	Scoring						
	Reporting	External Validity	Bias	Confounding	Power	Total	%
**Author(s) (Year)**	Items 1–10	Items 11–13	Items 14–20	Items 21–26	Item 27 *	Total/28	Percentage
**Bray et al., 2017** [26]	6	2	3	2	0	13	46.4%
**Easson et al., 2019** [17]	7	1	3	3	0	14	50%
**Thompson et al., 2021** [7]	7	0	2	2	1	12	42.9%
**Roberts et al., 2020** [18]	7	0	3	2	0	12	42.9%
**Zöller et al., 2017** [4]	8	1	3	3	0	15	53.6%
**Malins et al., 2018** [21]	7	1	4	1	0	13	46.4%
**Nomi et al., 2017** [16]	7	1	2	2	0	12	42.9%
**Mulligan et al., 2014** [23]	7	1	3	3	0	14	50%
**Zhang et al., 2016** [22]	8	1	3	2	0	14	50%
**Zöller et al., 2018** [30]	7	1	3	2	0	13	46.4%
**Dona et al., 2017** [24]	7	1	3	2	1	14	50%
**Jacobs et al., 2008** [28]	7	1	3	1	0	12	42.9%
**Seidel et al., 2020** [19]	8	0	3	3	1	15	53.6%
**Anderson et al., 2013** [20]	6	2	3	1	0	12	42.9%
**Dona et al., 2017** [25]	7	2	3	3	0	15	53.6%
**Wang et al., 2021** [10]	7	2	3	3	0	15	53.6%
**Zheng et al., 2021** [27]	7	2	3	3	0	15	53.6%
**Total**	120/187	19/51	50/119	38/102	3/27	230/486	47.3%
**%**	64.2%	37.3%	42.0%	37.3%	11.1%	47.3%	X

* Note: Modified Downs and Blacks checklist, 27 questions were used to score the risk of bias for each article, in each section, and to estimate an overall risk of bias. Item 27 was given no points if the study’s power was not reported. This table uses an “X” to identify a cell that cannot be filled with valid information.
